# Phase Composition and Its Spatial Distribution in Antique Copper Coins: Neutron Tomography and Diffraction Studies

**DOI:** 10.3390/jimaging7080129

**Published:** 2021-08-03

**Authors:** Bulat Bakirov, Irina Saprykina, Sergey Kichanov, Roman Mimokhod, Nikolay Sudarev, Denis Kozlenko

**Affiliations:** 1 Frank Laboratory of Neutron Physics, Joint Institute for Nuclear Research, 141980 Dubna, Russia; dolmen2002@mail.ru (I.S.); ekich@nf.jinr.ru (S.K.); denk@nf.jinr.ru (D.K.); 2 Institute of Physics, Kazan (Volga Region) Federal University, 420008 Kazan, Russia; 3 Institute of Archaeology RAS, 117036 Moscow, Russia; mimokhod@gmail.com (R.M.); sudarev@list.ru (N.S.)

**Keywords:** neutron imaging, neutron tomography, copper coins, Bosporan coins

## Abstract

The chemical and elementary composition, internal arrangement, and spatial distribution of the components of ancient Greek copper coins were studied using XRF analysis, neutron diffraction and neutron tomography methods. The studied coins are interesting from a historical and cultural point of view, as they are “Charon’s obol’s”. These coins were discovered at the location of an ancient Greek settlement during archaeological excavations on the “Volna-1” necropolis in Krasnodar Region, Russian Federation. It was determined that the coins are mainly made of a bronze alloy, a tin content that falls in the range of 1.1(2)–7.9(3) wt.%. All coins are highly degraded; corrosion and patina areas occupy volumes from ~27 % to ~62 % of the original coin volumes. The neutron tomography method not only provided 3D data of the spatial distribution of the bronze alloy and the patina with corrosion contamination inside coin volumes, but also restored the minting pattern of several studied coins. Taking into account the obtained results, the origin and use of these coins in the light of historical and economic processes of the Bosporan Kingdom are discussed.

## 1. Introduction

A detailed study of the chemical composition of ancient numismatic materials using structurally non-destructive diagnostic methods has gradually occupied its niche in the archeological natural-scientific research approaches [1,2,3]. The structural data obtained by these methods expand the possibilities of traditional studies of coins and provide knowledge about such aspects as the correlations of the nominal and metal value of coins [4,5], the identification of crisis periods [6], trade and political connections between ancient states and cultural groups [7,8], etc. Currently, coins are being intensely investigated by means of non-destructive physical methods, such as traditional techniques like metallography or X-ray diffraction [1,3,9]. In this context, we should also mention the neutron radiography [10,11,12] and neutron diffraction [12,13,14] methods as relatively modern structural non-destructive experimental approaches [1,15,16]. The fundamental difference in nature of neutron interaction with matter compared to X-rays provides additional benefits to neutron methods, including sensitivity to light elements, a notable difference in contrast between isotopes and high penetration effect through metals or heavy elements. Neutron imaging and scattering methods are successful in separating the components of coin alloys with neighboring elements: iron and nickel, copper and zinc [17], copper and silver [12,14], copper and lead [18]. Moreover, neutron structural methods were applied to determine the bulk composition hidden by corrosion [11,14] for coin identification [11], for the reconstruction of coinage technologies and sources of mining materials for coins [19] and for the description of coins’ degradation like internal corrosion tracks [11,20] and deterioration areas [18,21].

A great number of archaeological materials and unique finds, including a numismatic collection, was obtained by archeological excavations of the necropolis “Volna-1” [22,23], which is located 4 kilometers from the village Volna in the south-west of the Taman Peninsula around Mount Zelenskaya, Krasnodar region, Russian Federation. The necropolis burials date from the second quarter of the VI century BC to the beginning of the III century BC; the main period of its use was the second half of the VI-V centuries BC. The “Volna-1” necropolis [23,24] was presumably left by the Greek and barbarian population; the earliest burials of the necropolis may have been left by settlers who arrived from the territory of Great Greece. Numerous finds of Bosporan coins and Greek import items like tableware from Attica and Asia Minor, beads from North Africa, amphoras from the islands of the Aegean Sea and southern Pontus with wine and olive oil remains were indications of the well-being and life of the inhabitants of this ancient settlement. A bronze prosthesis with a wooden stopper for the leg, iron plate armor, a bronze Corinthian helmet of the “Hermione” type, musical instruments (kithara, lyre), a wreath on a gilded bone base with bronze petals and gold beads and other categories of archaeological items were excavated from the “Volna-1” archeological place [24]. 

A separate specific category of archeological items from the “Volna-1” necropolis are bronze coins. In total, as a result of excavations, nine coins were obtained. Almost everywhere their location was associated with the dead body: in the mouth, in the palm, at the elbow of the buried bodies. This is an indicator of a ceremony in the ancient Greek tradition, the so-called “Charon’s obol” [25,26]. The deceased person was accompanied by a coin, which he had to give to Charon, the ferryman of souls across the River Styx, which separates the world of the dead from the world of the living. Most of the found copper coins are covered with a thick layer of a patina, and their primary identification, the selection of the restoration procedure and the accurate study of their alloy composition are difficult. Here, we present neutron tomography and diffraction data supported by X-ray fluorescence analysis for the non-destructive identification of the copper alloy composition and for the reconstruction of the initial view of original coins and their remaining parts from under the patina layer.

## 2. Materials and Methods

### 2.1. The Coins Description

Photos of the reverse and obverse of the nine coins, the primary labeling and collection number of the ancient coins are presented in Table 1. The photos of the coins were obtained using a Leica M165 microscope with a video camera set-up. It can be seen that the surface of all of the coins is covered with a thick rough layer of patina, the minting pattern of the coins indistinguishable. The green color of the patina indicates a copper alloy.

### 2.2. X-ray Fluoresce Analysis

An analysis of the chemical composition of the patina on a coin surface was performed by the non-destructive X-ray fluorescence method on a portable spectrometer of the research class 5i Tracer (Bruker, Madison, WI, USA). The excitation source was an X-ray tube with power of 4 W and with a rhodium mirror. Voltage of 6–50 kV and a current range of 4.5–195 µA were automatically adjusted in the operating mode.

### 2.3. Neutron Diffraction

The phase composition of the volume of the coins was tested using the DN-12 neutron diffractometer [27] at the IBR-2 high-flux pulsed reactor (Frank Laboratory for Neutron Physics, JINR, Dubna, Russia). The neutron powder diffraction patterns were collected at the scattering angle of 2*θ* = 90°. The gauge volume covered the whole sample. The neutron diffraction patterns were analyzed by the Rietveld method using the Fullprof software [28]. The exposition time was 20 min. 

### 2.4. Neutron Tomography

The neutron tomography experiments were performed at the neutron radiography and tomography facility [29,30] at the IBR-2 high-flux pulsed reactor. A set of neutron radiography images was collected by a detector system based on a high sensitivity camera with a Hamamatsu CCD chip [15]. The tomography experiments were performed with a rotation step of 0.5°; the total number of measured radiography projections was 360. The exposure time for one projection was 20 s, and measurements were performed for 4 h in total. The imaging data were corrected by the camera dark current image and normalized to the image of the incident neutron beam using the ImageJ software [31]; the tomographic reconstruction was performed by the SYRMEP Tomo Project (STP) software [32]. Finally, a large data set containing a volume distribution of 3D pixels (voxels) were collected. The spatial resolution of the neutron tomography facility was 135 μm. The size of one voxel in our study was (52 × 52 × 52) μm^3^. The 3D volume data of voxels are the essence of the spatial distribution of values of the neutron attenuation coefficients inside the sample volume. Attenuation of the neutron beam corresponds to scattering and absorption losses inside the material [15], depending on the composition of the studied object. The VGStudio MAX 2.2 software of Volume Graphics (Heidelberg, Germany) were used for the visualization and analysis of reconstructed 3D data.

## 3. Results

### 3.1. The Composition of the Patina and Inner Volume of the Coins

The analysis of the chemical composition of the coin surfaces was performed without mechanical removal of the patina materials. It was found that all studied coins were made from copper-based alloys. The coins MP461, MP462, MP463, MP464, MP468, MP469 and MP467 were cast from low-tin bronze CuSn, two coins, MP465 and MP466, were cast of low-alloy triple bronze CuSnPb. At the same time, the lead on the surface of these two coins could be result of surface contamination.

The additional elements in the patina are lead in the concentration range of 0.11%–3.08%, tin of 2.59%–7.01%, arsenic of 0.11%–1.31%, antimony of 0.34%–1.12%, silver of 0.08%–0.20% and iron of 0.11%–0.25%. Nickel, zinc, cobalt, and other elements are present in very small quantities.

**Figure 1:**
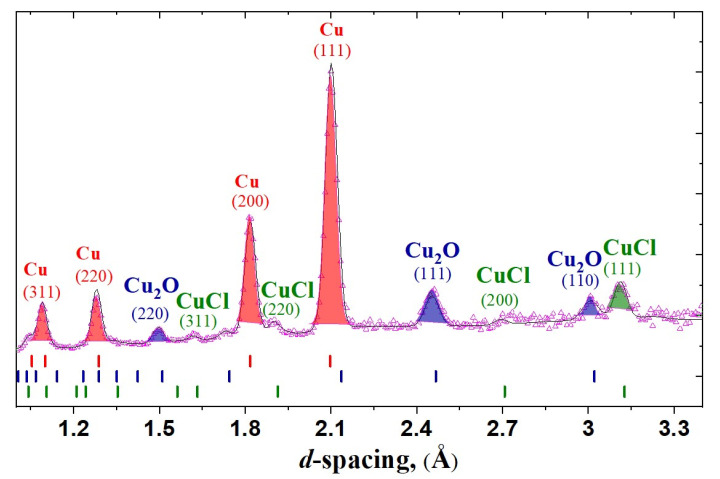
An example of the neutron diffraction patterns of coin MP467, for which the experimental points and fitted profile are shown. Tick marks are the calculated positions of the Bragg peaks of the copper, copper oxide and copper chloride phases. The most intense diffraction peaks of those phases with hkl-indexes are labeled.

The results of the chemical composition obtained from the surface of coins may differ from the distribution of chemical components in the entire coin volume [12,14]. The neutron diffraction method provides a non-destructive structural diagnostic with high penetration inside a volume of coins. As an example, the neutron diffraction pattern of the coin MP467 is shown in Figure 1. The analysis of all the neutron diffraction patterns of the studied coins provides data that the dominant phase of the coins is copper. The most intense diffraction peaks correspond to the copper cubic phase with $Fm\bar{3}m$ space group.

**Figure 2:**
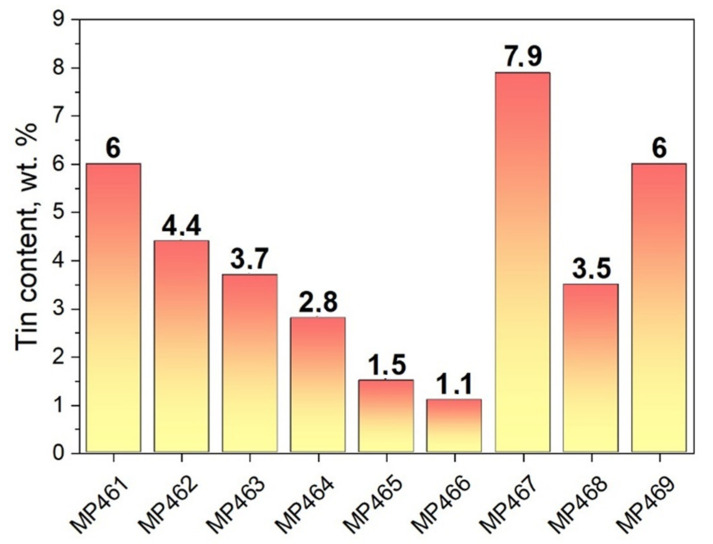
Diagram of the tin content in the studied bronze coins. The results were obtained from an analysis of neutron diffraction data. The average error in determining the tin content of bronze alloy does not exceed 0.3 wt.%.

It is known that, in a bronze alloy, tin atoms occupy the position of copper in the cubic crystal structure, and a disordered crystal solid solution is formed. The lattice parameter of the copper–tin cubic phase depends on the concentration of tin [33]. The parameters of the unit cell of the cubic phase of copper–tin alloy in consideration of pure copper lattice parameters, the corresponding values of the relative concentration of tin were calculated from the neutron diffraction data. The results obtained for bronze coins are shown in Figure 2. It can be seen that the tin content in the thickness of the coins falls in the range of 1.1–7.9%, which is consistent with the data of X-ray fluorescence analysis. It is interesting to note that a tin concentration of less than 2% was observed in the coins MP465 and MP466, which are characterized by the presence of lead (see Section 3.1). This may indirectly confirm the lead and tin content in the patina, or the contamination of the coins, rather than in their volume.

There are some additional diffraction peaks on the neutron pattern of the coins (Figure 1). We index these diffraction peaks as additional phases of cuprite Cu_2_O and copper chlorite CuCl phase.

### 3.2. Neutron Tomography

**Figure 3:**
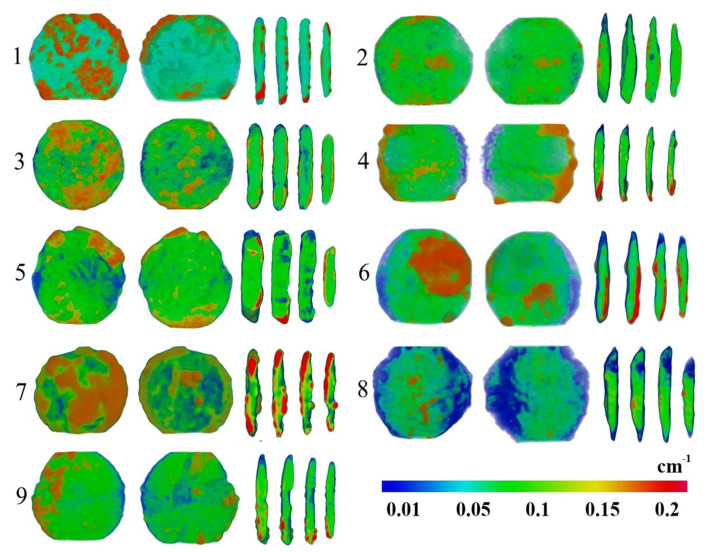
The 3D models after tomographic reconstruction and several transversal slices of the 3D models of the coins. The rainbow-like scale of neutron attenuation coefficients is shown. The regions with lower neutron attenuation coefficient can be attributed to copper–tin alloy areas, while the areas corresponding to high neutron attenuation coefficients are assumed to be corrosive contamination and patina materials. The dimensions of the reconstructed models correspond to the sizes of the coins shown in Table 1.

The spatial distribution of the internal components of the coins was studied using the neutron tomography method. The bronze alloy and corrosion-patina components form a good neutron radiographic contrast. The reconstructed 3D models and those virtual slices of the coins are presented in Figure 3. A great number of near-surface areas attributed to regions with higher neutron attenuation coefficients was observed. We believe those areas correspond to patina and corrosion contamination materials. From the neutron tomography data, we can calculate the total volume of each coin, separate the volume of the coin without the patina and surface contamination, then estimate the volumes of patina and contaminations. To quantify and compare the degree of degradation of studied coins, we used the segmentation algorithm described previously [34]. The direct determination of the boundary between the metal and patina regions is a nontrivial task [35]. In order to improve visual contrast, we used a special γ-filter [31] for tomography imaging data that allowed us to separate corresponding peaks in the total histogram and build an isosurface in the middle of the boundary between the high and low values of the gray-shade scale. Then, the isosurface was geometrically translated to the initial 3D data using the calculated boundary conditions for the virtual separation process. A segmentation procedure with the same boundary conditions was performed for all studied coins. This algorithm does not result in absolute values of the volumes of patina or the metal base of coins, but the segmentation procedure is suitable for comparative analysis and estimated calculations. The results of the analysis of 3D data are presented in Table 2.

From the obtained data of Table 2, the ratio of the volume of the copper alloy to the total volume of coins, as a structural indicator of the degree of the degradation of coins, can be estimated. The degradation due to the corrosion and patina expansion of studied coins can vary from 27% of the degradation in volume for the MP461 coin to 62% of the volume for the MP468 coin. The average degradation level of all the studied coins is about 35%. The high degree of degradation of all coins does not allow one to restore the minting pattern on the obverse or reverse, or to accurately identify the studied coins for historical interpretation. However, after using several filter procedures and analyzing algorithms for three-dimensional neutron data, we were able to restore several elements of the coinage on the surface of the copper alloy volume.

**Figure 4:**
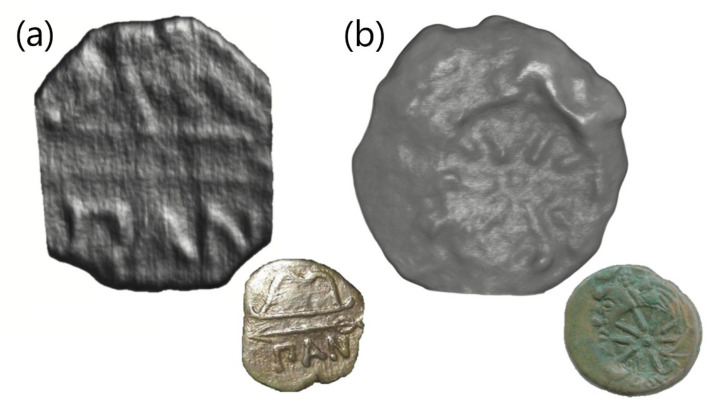
The examples of reconstructed patterns on the surface of several coins from the neutron tomography data. (**a**) The MP468 coin with patterns of a bow, arrow and the inscription “IIAN”. (**b**) The reconstructed view of the “Twenty-ray star” on the MP465 coin. A photo of a similar coin from the catalog [36,37].

Methods of improving the visualization of the restored three-dimensional model of the least degraded coin, MP468, allowed us to accurately identify the words “IIAN”, identify the remnants of an image of a bow and a straight line as the image of an arrow (Figure 4a) [21,36]. This pattern indicates the Panticapean obol. Panticapaeum was an ancient Greek city founded at the end of the VII century BC by settlers from Miletus on the location of modern Kerch, Crimea. The Panticapean copper coins played a very significant role in the monetary circulation of the Bosporan Kingdom [21,22]. The copper coins of the Panticapaeum with the observed coinage pattern are attributed to the first quarter of the IV century BC of the period around 275–250 BC. 

A round-shaped «Twenty-ray Star» stamp [36,37] was found on the coins MP465 and MP469 (Figure 4b). Similar patterns have been found on a great number of copper coins previously [35]. «Twenty-ray Star» stamps are indicative of the re-minting of initial copper Panticapean coins. Researchers and specialists agree that the re-minting or additional stamping of copper coins is related to a change of coin nominee or value [21,38]. This process has been called the “crisis of the Bosporus coin minting” [34]. The characteristic features of the crisis were frequent issues and changes in the coin types, changes in denominations, re-minting or over-minting [37]. However, discussion about the increasing or reducing of the value of the original coin after re-minting is still ongoing [39]. Within the limits of this work, we can only assume that the initial tetrachalcon [21], as the Panticapean copper coin, double increased its nominal value to obol [39].

## 4. Conclusions

We present the results of non-destructive studies of the several bronze coins as interesting representatives of ancient cult objects, “Charon’s obols”. These coins were found in the burials of a large necropolis, “Volna-1”, during archeological works and were the payment of the souls to Charon, the ferryman across the River Styx. The high degree of degradation of up to 62% of the volume does not allow us to accurately identify the type and origins of these coins. The high penetrating feature and the nature of the interaction of neutrons with matter allowed us to determine the phase composition of the coin volumes, as well as to separate coin alloys and patina materials. The neutron tomography results provide partial restoration of the elements of the minting patterns of these coins. The structural and imaging neutron data allowed us to provide some conclusions about the origins of the found coins and their monetary circulation period.

## Figures and Tables

**Table 1:** The photo and labeling of studied coins.

N	Collection Label	Photos of Obverse and Reverse of the Coins
1	MP461	** 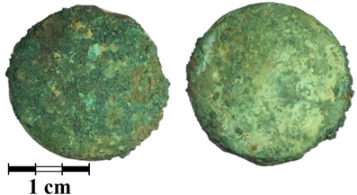 **
2	MP462	** 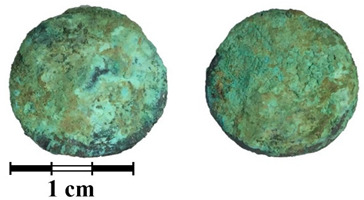 **
3	MP463	** 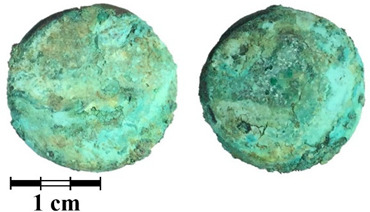 **
4	MP464	** 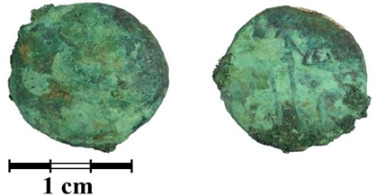 **
5	MP465	** 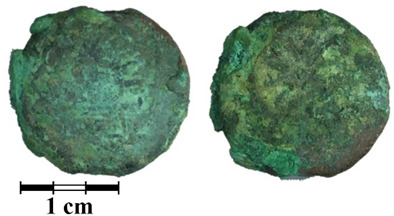 **
6	MP466	** 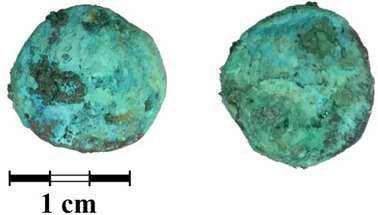 **
7	MP467	** 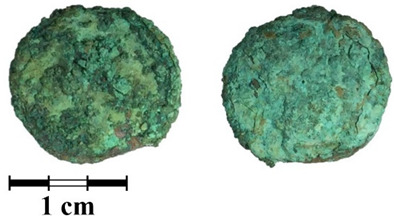 **
8	MP468	** 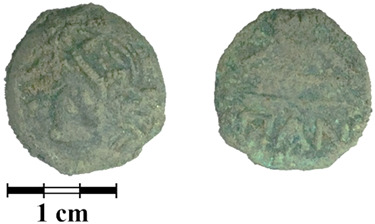 **
9	MP469	** 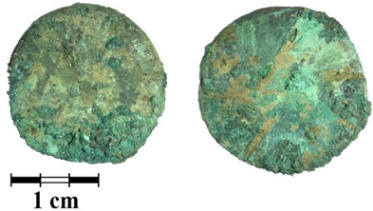 **

**Table 2:** The volumes of the coins, copper–tin alloy and patina and surface contaminations obtained from neutron 3D data analysis.

Coin	Total Coin Volume, mm^3^	Alloy Volume, mm^3^	Patina Volume, mm^3^	Volume of Surface Contaminations, mm^3^
MP461	615.9(3)	449.2(2)	95.2(2)	71.5(2)
MP462	204.5(2)	153.6(3)	48.6(2)	2.3(2)
MP463	898.1(3)	745.5(2)	102.3(2)	50.3(2)
MP464	139.9(2)	78.5(2)	53.9(2)	7.5(1)
MP465	746.8(3)	607.7(2)	77.8(2)	61.4(2)
MP466	226.7(2)	138.2(2)	57.3(3)	31.2(3)
MP467	375.9(2)	225.1(3)	56.8(2)	94.0(3)
MP468	346.7(2)	131.0(2)	212.1(3)	3.6(2)
MP469	576.2(3)	419.6(2)	114.0(3)	42.6(2)

## Data Availability

No new data were created or analyzed in this study. Data sharing is not applicable to this article.
